# Supplementary material for: MetaRibo-Seq measures translation in microbiomes
Source: Nat Commun. 2020 Jun 29;11:3268. doi: 10.1038/s41467-020-17081-z (PMC7324362; doi:10.1038/s41467-020-17081-z)
Supplement: Supplementary file 10 — Supplementary Data 7 [file 41467_2020_17081_MOESM10_ESM.zip › File2/Confidence_High_Taxonomy/221807_out.krona.html]

Javascript must be enabled to view this page.

members
magnitude
magnitudeUnassigned
count
unassigned
taxon
rank

221807\_out

17

2
superkingdom
17

phylum
1
32066

1
class
203490

203491
order
1

family
1
203492

genus
1
848

species
1
851


SRS024447\_contig\_number\_8970
76857
1
subspecies

phylum
16
976

16
class
117743

16
order
200644

family
16
49546

1

SRS893328\_contig\_number\_7653
10
genus
59732

2
species

SRS012922\_contig\_number\_15900SRS075353\_contig\_number\_29290
1500286

1736366

SRS015692\_contig\_number\_16560SRS893385\_contig\_number\_33791
species
2

2
species

SRS015278\_contig\_number\_12279SRS098824\_contig\_number\_18934
2487065

1736361

SRS020406\_contig\_number\_9579
species
1

421072

SRS104320\_contig\_number\_35355
species
1

1721091

SRS098616\_contig\_number\_contig-100\_5395.91412
species
1

1016
genus
6

2
species

SRS075959\_contig\_number\_8242SRS149237\_contig\_number\_11432
1017

1019

SRS053584\_contig\_number\_7119SRS077283\_contig\_number\_17176
species
2


SRS077312\_contig\_number\_22736
1227265
1
species

1
species

SRS019906\_contig\_number\_4443
209053
